# Depression, inflammation, and memory loss among Mexican Americans: analysis of the HABLE cohort

**DOI:** 10.1017/S1041610217001016

**Published:** 2017-06-20

**Authors:** Leigh A. Johnson, Melissa Edwards, Adriana Gamboa, James Hall, Michelle Robinson, Sid E. O'Bryant

**Affiliations:** 1 Center for Neuroscience Discovery, University of North Texas Health Science Center, Fort Worth, Texas, USA; 2 Institute for Aging & Alzheimer's Disease Research, University of North Texas Health Science Center, Fort Worth, Texas, USA; 3 Department of Psychiatry, University of North Texas Health Science Center, Fort Worth, Texas, USA; 4 Texas College of Osteopathic Medicine, Fort Worth, Texas, USA; 5 Boehringer Ingelheim pharmaceuticals, Ridgefield, Connecticut, USA

**Keywords:** depression endophenotype (DepE), Mexican-Americans, mild cognitive impairment

## Abstract

**Background:**

This study explored the combined impact of depression and inflammation on memory functioning among Mexican-American adults and elders.

**Methods:**

Data were analyzed from 381 participants of the Health and Aging Brain study among Latino Elders (HABLE). Fasting serum samples were collected and assayed in duplicate using electrochemiluminesce on the SECTOR Imager 2400A from Meso Scale Discovery. Positive DepE (depression endophenotype) was codified as any score >1 on a five-point scale based on the GDS-30. Inflammation was determined by TNFα levels and categorized by tertiles (1st, 2nd, 3rd). WMS-III LMI and LMII as well as CERAD were utilized as measures of memory. ANOVAs examined group differences between positive DepE and inflammation tertiles with neuropsychological scale scores as outcome variables. Logistic regressions were used to examine level of inflammation and DepE positive status on the risk for MCI.

**Results:**

Positive DepE as well as higher inflammation were both independently found to be associated with lower memory scores. Among DepE positive, those who were high in inflammation (3rd tertile) were found to perform significantly worse on WMS-III LM I (F = 4.75, p = 0.003), WMS-III LM II (F = 8.18, p < 0.001), and CERAD List Learning (F = 17.37, p < 0.001) when compared to those low on inflammation (1st tertile). The combination of DepE positive and highest tertile of inflammation was associated with increased risk for MCI diagnosis (OR = 6.06; 95% CI = 3.9–11.2, p < 0.001).

**Conclusion:**

Presence of elevated inflammation and positive DepE scores increased risk for worse memory among Mexican-American older adults. Additionally, the combination of DepE and high inflammation was associated with increased risk for MCI diagnosis. This work suggests that depression and inflammation are independently associated with worse memory among Mexican-American adults and elders; however, the combination of both increases risk for poorer memory beyond either alone.

Mexican-Americans often experience significant health disparities compared to non-Hispanic whites. As the fastest aging segment of the US population, Mexican-Americans face a significant health disparity when it comes to the development of neurodegenerative diseases. Mild cognitive impairment (MCI), considered as a prodromal category of neurodegenerative diseases, has been shown to be particularly high among Mexican-Americans (Dubious *et al*., 2007; Teixeria *et al*., 2012; O'Bryant *et al*., 2013a; 2013b; Alzheimer's Association, 2014). Risk factors for developing MCI among this population have been found to include diabetes, depression, and obesity (O'Bryant *et al*., 2013b; Johnson *et al*., 2015b; Downer *et al*., 2016). In our work, Mexican-Americans were found to have a higher prevalence of depressive symptoms (O'Bryant *et al*., 2007; O'Bryant *et al*., 2013b; O'Bryant *et al*., 2013c) and these symptoms were associated with worse cognitive functioning when compared to non-Hispanic whites. While some risk factors for neurodegenerative disease cannot be changed, depression has often been considered a modifiable risk factor for the development of MCI. Therefore more research is needed to understand the relationship between depression and memory loss in this underserved, yet burgeoning segment of the aging population.

Deconstructing the relationship between depression and memory may be hindered by our treatment of depression as a unitary construct. Our group has proposed that we must understand the influence of the impact of specific depressive symptoms on cognition. Our research team identified and validated a depressive endophenotype of cognitive impairment, called DepE (Johnson *et al*., 2013). Individuals with elevated DepE scores were at increased risk for MCI and Alzheimer's disease (AD) diagnoses, and demonstrated significantly poorer neurocognitive abilities at baseline and follow-up assessments. DepE was also tested in a cohort of cognitively normal cohort of adults from the Western Australian Memory Study, which found elevated DepE was associated with poorer cognitive performance among healthy cognitively normal individuals (Johnson *et al*., 2013).

Inflammatory processes have been proposed as a major component of the pathogenesis of both depression and dementia. Changes in serum acute-phase proteins, chemokines, adhesion molecules, and pro-inflammatory cytokines have all been implicated in playing a role in the development of major depression. Increases in tumor necrosis factor-α (TNFα), interleukin 6 (IL6), and c-reactive protein have been consistently linked with increased depressive symptoms (Pucak and Kaplin, 2005; Simen *et al*., 2006; Losleben-Berthold and Himmerich, 2008; Miller *et al*., 2009; Trollor *et al*., 2012; Huang and Lin, 2015). TNFα concentrations have been found to be increased in depressed females as compared to healthy controls (Kahl *et al*., 2006). TNFα concentrations have also been linked to poorer cognition (Jefferson *et al*., 2011; Trollor *et al*., 2010) and the development of Alzheimer's (Buchhave *et al*., 2010; McCaulley and Grush, 2015). Windham *et al*. (2014) found a strong relationship between cognition and soluble TNF receptors 1 and 2 especially for African-Americans.

The relationship between TNFα, cognition, and depression has not been extensively investigated. Matsushima *et al*. (2015) found no relationship between TNFα and cognition and depression in a small sample of community-dwelling cognitively normal Japanese elderly. Lindqvist *et al*. (2013) in a study of Parkinson's patients using CSF inflammatory markers found no relationship between TNFα, depression, and MMSE scores. Although these studies indicate little relationship, the pathophysiology of TNFα effecting cognition and mood suggests that a relationship may be found especially in populations where both depression and inflammatory diseases are prevalent, such as found for Mexican-Americans. In our work, inflammation was associated with diagnosis of MCI among Mexican-Americans (Edwards *et al*., 2015). The current study sought to examine the additive impact of elevated inflammation to DepE on memory abilities and risk for MCI diagnosis among community-dwelling Mexican-American adults and elders.

## Methods

### Participants

Data from 381 participants from the Health and Aging Brain among Latino Elders (HABLE) study were analyzed (Edwards *et al*., 2015; Johnson *et al*., 2015a; Szerlip *et al*., 2015). The HABLE study is an ongoing epidemiological study of cognitive aging among community-dwelling Mexican-American individuals. The HABLE study utilizes a community-based participatory research (CBPR) approach, which is a research methodology that involves partnering communities with scientific groups to conduct studies of human disease. The generation of locations for targeted CBPR recruitment was determined through analysis of zip codes in Tarrant County with the highest population density of Hispanic individuals. This research was conducted under an IRB 2012-O83 approved protocol with each participant (and/or informants for cognitively impaired persons) providing written-informed consent.

### Study design

Each participant underwent an interview (i.e. medical history, medications, and health behaviors), detailed neuropsychological testing, blood draw, and medical examination (review of systems, Hachinski Ischemic Index scale, brief neurological screen). The neuropsychological battery consisted of tests of executive functioning: Trail Making Test, EXIT25, clock drawing (Sunderland *et al*., 1989; Royall *et al*., 1992; Strauss *et al*., 2006); language: FAS and Animal Naming (Strauss *et al*., 2006); visuospatial skills: CLOX2 (Sunderland *et al*., 1989); memory: (Wechsler Memory Scale – 3rd ed. (WMS-3) Logical Memory (Weschler, 1987) and Consortium for the Establishment of Registry for Alzheimer's Disease List Learning (Morris *et al*., 1989), and attention: WMS-3 Digit Span (Weschler, 1987). Testing was completed in English or Spanish depending on the participant's preference. Here, analyses focused specifically on WMS-3 Logical Memory and CERAD List Learning scores. The DepE score was derived from the Geriatric Depression scale, which was also administered to all participants (Yesavage *et al*., 1982). Age and education corrected scale scores were utilized for analyses. The current team has generated normative references for each of these tests for English- and Spanish-speaking Mexican-Americans for diagnostic purposes (manuscripts in preparation). Cognitive diagnoses of MCI were assigned according to Mayo Clinic criteria (Peterson, 2003). Pre-MCI was defined as those who fell one standard deviation below the mean on CERAD List Recall but for whom were classified as cognitively normal. All diagnoses were determined through a consensus review panel.

### Blood collection and processing

Blood collection and processing was conducted per international guidelines (O'Bryant *et al.*, 2015). Briefly, fasting blood was collected as follows: (1) venipuncture using 21 g needle, (2) sample tubes collected in the following order – blood culture tube, coagulation tube, serum, heparin, plasma EDTA tube, (3a) serum tube will be allowed clot for 30 minutes at room temperature in a vertical position, (3b) plasma tubes gently inverted 5–10 times, (4) centrifuged with horizontal rotor for 10 minutes at 2,000 × g within one hour of collection, (5) 1.0-mL aliquots of serum will be transferred into polypropylene (cryovial) tubes, (6) sample ID (Freezerworks™ barcode labels) affixed to each aliquot, and (7) samples will be placed into −80 °C freezer within two hours of collection. Serum TNFα was assayed in duplicate via a multi-plex biomarker assay platform using electrochemiluminesce (ECL) on the SECTOR Imager 2400A from Meso Scale Discovery (MSD; http://www.mesoscale.com) per our previously published methods (O'Bryant *et al.*, 2013a). ECL measures have well-established properties of being more sensitive and requiring less sample volume than conventional ELISAs (Kuhle *et al*., 2010), the gold standard for most assays.

### Statistical analyses

A positive score on DepE was codified as any score >1 on the five-point scale. TNFα levels were categorized by tertile scores (1, 2, or 3). Group differences by DepE positive (>1) and TNFα tertiles were examined by ANOVA with age and education corrected neuropsychological scale scores utilized as outcome variables. Additionally, ANCOVA analyses were conducted to verify the analyses with age, gender, and education used as covariates with the results remaining the same. Risk for MCI diagnosis by DepE positive and TNFα third tertile was examined via logistic regression.

## Results

Demographic characteristics of the sample by DepE scores and TNFα tertiles are presented in Table 1. As can be seen, in the DepE positive group those in the third tertile of TNFα were significantly older than those in the 1st tertile. There were no significant differences in educational attainment. Among the DepE negative group, those in the 3rd tertile of TNFα were significantly older than those in the 1st tertile. There were no significant education discrepancies.

**Table 1.** Demographic characteristics

	depe positive				depe negative			
	tnfα	tnfα	tnfα		tnfα	tnfα	tnfα	
	tertile 1	tertile 2	tertile 3		tertile 1	tertile 2	tertile 3	
	n = 67	n = 53	n = 62	p-value	n= 57	n = 74	n = 79	p-value
Age	58.6(7.1)	61.0 (7.9)	63.2(8.3)	1vs2 NS	59.0(6.6)	60.1(9.2)	62.7(7.9)	1vs2 NS
				1vs3 p = 0.001				1vs3 p = 0.006
				2vs3 NS				2vs3 NS
Education	7.3(3.6)	6.1(3.3)	6.6(3.8)	NS	9.0(4.7)	9.0(4.7)	7.7(4.3)	NS
Gender	90%	80%	71%		65%	80%	68%	
(% female)								

NS = no significant.

As can be seen from Table 2, those who are DepE positive score worse on memory measures than those who are DepE negative, which is consistent with our prior work (Johnson *et al*., 2013; Johnson *et al*., 2015a). When looking within DepE categories, increasing level of inflammation was associated with worse memory performance (see Table 2).

**Table 2.** Mean memory scale scores by DepE and TNFα levels

	depe positive				depe negative			
	tnfα	tnfα	tnfα		tnfα	tnfα	tnfα	
	tertile 1	tertile 2	tertile 3		tertile 1	tertile 2	tertile 3	
	n = 67	n = 53	n = 62	p-value	n = 57	n = 74	n = 79	p-value
WMS III LM1	10.3(3.1)	9.0(3.7)	8.5(3.7)	1vs2 p = 0.04	11.1(2.8)	10.7(3.2)	9.4(3.8)	NS
				1vs3 p = 0.003				
WMS III LM2	10.2(3.1)	8.6(3.5)	7.7(3.8)	1vs2 p = 0.01	11.3(2.9)	11.0(3.2)	9.4(3.8)	1vs2 NS
				1vs3 p < 0.001				1vs3 p = 0.001
								2vs3 p = 0.004
CERAD	9.2(2.6)	8.0(2.7)	6.4(2.8)	1vs2 p = 0.02	8.5(2.6)	8.6(2.6)	7.6(3.4)	1vs2 NS
List Recall				1vs3 p < 0.001				1vs3 p = 0.08
				2vs3 p = 0.002				2vs3 p = 0.03

NS = no significant.

Within the DepE positive group, those in the 3rd tertile of TNFα performed worse on all measures. Specifically, those in the 3rd tertile of TNFα scored significantly worse than those in the 1st tertile on WMS-III Logical Memory 1 (p = 0.003), Logical Memory 2 (p < 0.001), and CERAD List Learning (p < 0.001). Those in the 2nd tertile of TNFα performed significantly worse on WMS-III Logical Memory 1 (p = 0.04), WMS-III LM 2 (p = 0.01), and CERAD List Recall (p = 0.02). Those in the 3rd tertile of TNFα also performed significantly worse than those in the 2nd tertile on the CERAD List Recall (p = 0.002). In the ANCOVA analyses, the results remained the same.

Within the DepE negative group, those participants in the 3rd tertile generally performed worse on memory tests than the other groups as well, although the findings were not as stark as among the DepE positive group. Those in the 3rd tertile of TNFα performed significantly worse than the 1st tertile on the WMS-III LM2 (p = 0.001). Those in the 3rd tertile of TNFα also performed significantly worse than those in the 2nd tertile on the WMS-III LM2 (p = 0.004) and CERAD (p = 0.03).

In the logistic regression, being DepE positive + TNFα 3rd tertile was associated with significant risk for MCI diagnosis (OR = 6.06, 95% CI = 3.9–11.2).

## Discussion

This study examined the impact of inflammation and depression on memory among Mexican-American adults and elders. The results indicated that those with positive DepE scores and higher levels of inflammation performed poorer on all memory measures when compared to those with low inflammation. Furthermore, individuals with both positive DepE and placement in the highest inflammation tertiles were found to be at increased risk for MCI. The results suggest that inflammation may (1) play a key role in the relationship between depression and memory and/or (2) have an additive impact on memory decline when found in conjunction with depression.

Proinflammatory cytokines, such as TNFα, have been implicated in the pathophysiology of dementia and depression. Past research studies have found higher concentrations of TNFα were found in depressed patients as compared to healthy controls (Dowlati *et al*., 2010) and inflammation has been consistently linked to cognitive dysfunction, MCI, and AD in our prior work (O'Bryant *et al*., 2014; Edwards *et al*., 2015). However, studies examining the relationship between inflammatory markers, memory, and depression have been mixed. Several studies have found no relationship between TNFα and cognition and depression (Matsushima *et al*., 2015). Himmerich *et al.* (2008) suggested these inconsistencies may be due to the subgroups of depression. It is possible that a specific subtype of depression is linked to inflammatory cytokines. This study is one of the first to examine the role of inflammation in a subgroup of depressed elders.

There are limitations of the current study. First, the current study utilized a cross-sectional design thus casual inferences may not be drawn for the results. Future research should examine the role of inflammation on the depression-cognition link utilizing a longitudinal design. Our group is currently collecting follow up data to examine this finding over time. Second, the current sample is insufficient to determine the relative contribution of inflammation or depression on cognition. Our group has received funding to increase the sample size of this cohort and we hope to be able to address this in future work. Next, this study examined only TNFα levels. Future work should look at additional markers of inflammation to examine profiles that may further clarify this link. The focus on this study was on memory, however, future work should examine the impact of this relationship on other cognitive domains. Cognitive diagnoses were assigned via consensus review, and therefore not confirmed via PET scan or lumbar biomarker analyses. Thus, it is possible the MCI cases are not pre-AD, as data on amyloid biomarkers was not available for this study. A significant advantage to this work; however, is that participants from this study were recruited from a community-based sample of elders rather than a clinic. Another significant advantage to this study is the sample size and the sample size within each subgroup is greater than 50. Last, this study utilized an underserved and understudied population. By 2050, the number of Hispanics in America over age 65 will nearly triple (Jacobsen *et al*., 2011), with Mexican-Americans making up over 65% of this group. Since advancing age is the greatest risk factor for developing cognitive impairment, Mexican-Americans will be disproportionately impacted by MCI/AD. Despite these facts, there are only a few aging research studies focusing on Mexican-Americans.

This study is the first to specifically examine the impact of inflammation on depression and memory among Mexican-American adults and elders. Utilizing the sub-group approach to classifying depression may help to elucidate the role inflammation plays in depression. Our past research has focused on identifying a subset of elders that experience depression related cognitive loss, and thus can accurately identify a selective group that may benefit from antidepressant treatment for prevention of memory loss and AD. These findings indicate that those individuals with elevated TNFα, are at great risk for memory loss, which may suggest a specific combination therapy approach to preventing and treating memory loss among this specific subgroup of Mexican-American older adults. Further work is warranted to continue to push forward this notion of precision medicine that is tailored to specific ethnic groups, which is akin to ongoing efforts in the cancer, diabetes, and cardiovascular disease spaces. While this work focused specifically on memory loss, the current team is expanding the analyses to examine the impact of depression and inflammation on other cognitive domains, including executive functioning, language, attention/processing, and visuospatial abilities

## Conflict of interest

None.

## Authors’ contributions

Conceived and designed the experiments: LAJ, AG, ME, JH, MR, SO. Performed the experiments: LAJ, AG, ME, JH, MR, SO. Analyzed the data: LAJ, AG, ME, MR, JH, SO. Contributed reagents/materials/analysis tools: LAJ, AG, ME, MR, JH, SO. Wrote the manuscript: LAJ, AG, MR, ME, JH, SO. Other: ICMJE criteria for authorship read and met: LAJ, AG, ME, MR, JH, SO. Agree with manuscript results and conclusions: LAJ, AG, ME, MR, JH, SO.

## List of abbreviations

CBPR, community-based participatory research
CERAD, Consortium for the Establishment of Registry for Alzheimer's Disease
DepE, depression endophenotype
ECL, electrochemiluminesce
HABLE, Health and Aging Brain study among Latino Elders
IL6, interleukin 6
LM, logical memory
MCI, mild cognitive impairment
TNFα, tumor necrosis factor-α
WMS-III, Wechsler Memory Scale – 3rd ed.
